# Comprehensive Evaluation of Body Lotion in Alleviating Xerosis: A Multi‐Omics Approach to Lipid Metabolism and Microbial Community Modulation

**DOI:** 10.1111/jocd.70711

**Published:** 2026-02-01

**Authors:** Jun Wang, Lu Cheng, Jiaqi Zhang, Yi Qin, Qitian Fu, Xiaofeng Bai, Fengwei Qi, Fan Wu, Jie Yang, Yao Pan

**Affiliations:** ^1^ Department of Cosmetics, School of Light Industry Science and Engineering Beijing Technology and Business University Beijing China; ^2^ Beijing Key Laboratory of Plant Research and Development Beijing China; ^3^ Shandong Huawutang Biotechnology Co., Ltd. Jinan China

**Keywords:** lipidomics, microbiomics, moisturizers, skin barrier, xerosis

## Abstract

**Background:**

Xerosis, marked by a compromised skin barrier and disrupted lipid metabolism, leads to dryness, scaling, and itching. Ceramide and natural oil‐based moisturizers can improve skin hydration and barrier repair, but their effects on lipid networks and microbiome interactions have not been well understood.

**Methods:**

A multicenter, randomized, self‐controlled study was conducted to assess the efficacy of a body lotion formulated with ceramides and natural oils in the management of xerosis. The lotion was applied daily to one leg for 4 weeks, with the other leg as a control. Skin radiance, skin scaliness, skin smoothness, stratum corneum hydration, transepidermal water loss, and pH were measured at various intervals. Lipidomics and microbiomics analyses evaluated changes in lipid metabolism and microbial structure.

**Results:**

The body lotion enhanced skin hydration, radiance, and smoothness, while decreasing TEWL and scaling. Lipidomics showed higher levels of essential lipids in the treatment group. Microbiome analysis revealed increased diversity, with more *Firmicutes* and *Cutibacterium* and less *Proteobacteria*, indicating improved skin barrier and microbial balance.

**Conclusion:**

This body lotion effectively alleviates dryness, significantly improving skin hydration, barrier function, and texture. It achieves these benefits by restoring the skin's lipid balance and optimizing the microbial community, with lipid‐microbiome crosstalk identified as a key mechanism. This multi‐omics insight provides a foundation for the targeted management of dry skin.

## 1 Introduction

Xerosis is a common dermatological condition primarily associated with impaired skin barrier function, and it is characterized by clinical manifestations such as skin roughness, scaling, pruritus, and fissuring [1]. This condition exhibits seasonal variability, with increased prevalence and severity during the winter months because of low humidity and environmental dryness [2]. Xerosis predominantly manifests on the lower extremities, particularly the shins and calves, where the density of sebaceous glands is minimal and transepidermal water loss is pronounced [3]. This condition is characterized by compromised skin barrier function, and its pathogenesis is closely linked to reduced synthesis of lipids, especially ceramides [4]. This metabolic disturbance directly results in structural disruption of the stratum corneum (SC), depletion of natural moisturizing factors (NMFs), and ceramide deficiency, thereby causing persistent pruritus and significantly decreasing patient quality of life [5].

Skin lipids function as the primary physical barrier within the “brick‐and‐mortar” architecture, where corneocytes represent the bricks and lipids act as the mortar. Additionally, they play a crucial role in the chemical barrier by modulating antimicrobial peptide activity, thus ensuring barrier homeostasis [6]. The skin microbiota further enhances barrier protection by competing for nutrients, secreting antimicrobial compounds such as short‐chain fatty acids, and modulating the immune response [7]. Importantly, when the barrier is compromised, there is a reduction in microbial diversity and an increase in pathogen colonization, such as by *Staphylococcus aureus* , which perpetuates a cycle of barrier disruption and heightened inflammation [8].

Moisturizers play a fundamental role in managing xerosis, serving as a foundational component of long‐term dry skin management, highlighting their preventive and reparative benefits in maintaining cutaneous homeostasis [9]. Peony seed oil, *Hippophae rhamnoides* seed oil, palm kernel oil, and shea butter are natural oils and key moisturizer ingredients known for their dermatological benefits, providing deep hydration and antioxidant protection [10, 11]. As the major component of human skin lipids, ceramide‐related components such as ceramide NP, NS/NG, AP, and EOP are vital for maintaining the skin barrier and improving hydration, reducing water loss [12, 13]. Evidence has demonstrated that twice‐daily application of moisturizers significantly improves skin hydration and barrier function across all age groups, helping to minimize discomfort and mitigate complications associated with severe dryness [14, 15]. However, these studies mainly target objective measures and symptom scores after moisturizer treatment and do not explore the mechanism underlying lipid metabolism changes and microbiota effects.

We employ a randomized, self‐controlled, multicenter, and prospective study to systematically assess the moisturizing and repair efficacy of a body lotion through a multidimensional approach. In addition to biophysical parameter measurement and clinical assessment, this study incorporates lipidomics and microbiomics for the first time to elucidate the regulatory effects of moisturizers on lipid metabolism networks and microbial community structures. This study aims to elucidate the molecular mechanisms underlying the action of moisturizers and offers a theoretical foundation for their precise clinical application.

## 2 Materials and Methods

### 2.1 Test Product

The tested body lotion was obtained from Shandong Huawutang Biotechnology Co., Ltd. (Shandong, China). The body lotion contained *Paeonia suffruticosa* seed oil, Ceramides NP, NS/NG, AP, and EOP, *Butyrospermum parkii* (shea) oil, *Elaeis guineensis* (palm) kernel oil, and *Hippophae rhamnoides* seed oil as the main functional ingredients. The subjects were instructed to apply the body lotion to their lower leg one time a day for 4 weeks.

### 2.2 Subjects

The subjects in this research were healthy adults aged 18–45 years who all had mild to moderate dryness on both lower legs. The European Cosmetics Effectiveness Evaluation Expert Group's (EEMCO) Overall Dry Skin Score (ODS) was used to thoroughly assess the degree of skin dryness [16, 17]. The exclusion criteria were as follows: (i) individuals who had or were currently receiving physical therapy or medication for any systemic or skin disease within the past 3 months; (ii) highly sensitive individuals or individuals allergic to known product ingredients; and (iii) individuals who were pregnant, breastfeeding, or were intending to conceive soon.

The study was conducted in accordance with the principles of the Declaration of Helsinki.

### 2.3 Study Design

Each participant's left and right lower legs were allocated to either the treatment or control group in this noninvasive, randomized controlled study. Subjects were instructed not to use any other skin care products or alter their body washing routine (product or frequency) from 1 week before the trial began until the completion of the study. The participants were told to use the body lotion once daily to one side of their lower leg as the treatment group and to avoid using any other product on the other leg as the control group for 4 weeks after this 7‐day run‐in phase. The following skin parameters were examined at baseline (BL), two hours (2 h), twenty‐four hours (24 h), two weeks (W2), and four weeks (W4) of each phase: skin radiance, skin scaliness (SEsc), skin smoothness (SEsm), stratum corneum hydration (SCH), transepidermal water loss (TEWL), and pH. To avoid environmental impacts, the subjects washed their lower legs with water and were acclimated to the testing room at a temperature of 22°C ± 1°C and a relative humidity of 50% ± 5% for 30 min before the skin tests were performed. Additionally, at baseline and 4 weeks after treatment, skin microbiota and skin lipid samples were taken.

### 2.4 Skin Physiological Measurements

A Corneometer CM 825 probe (Courage & Khazana, Koln, Germany) was used to assess skin SCH, and a Tewameter TM300 (Courage & Khazana, Koln, Germany) was used to measure skin TEWL. Radiance was measured with a Glossymeter GL200 (Courage & Khazaka, Germany). A Skin pH meter PH 905 probe (Courage & Khazana, Koln, Germany) was used to test the pH of the skin. To evaluate the surface characteristics of the skin, a Visioscan VC20plus (Courage & Khazana, Koln, Germany) was used to perform a surface evaluation of the living skin (SELS) parameters. These parameters included SEsc and SEsm. All skin parameters except SCH (6 times) were recorded as the average of three repeated tests.

### 2.5 Skin Lipid Sampling and Analysis

Sebutape test strips were applied to one side of each subject's lower legs by the researcher. To eliminate potential contamination from residual lotion on the skin surface, the first tape strip was discarded. A second test strip was then applied with standardized pressure, removed after 3 min, and collected for analysis. The samples were then stored at −80°C. Lipids were extracted using the Bligh and Dyer method with methanol. Lipids were chromatographically separated via ultra‐performance liquid chromatography–quadrupole time‐of‐flight mass spectrometry (UPLC–QTOF–MS). High‐resolution QTOF–MS provided accurate mass–to–charge (*m*/*z*) ratios for lipid identification [18].

In reference to the methodology in the literature, principal component analysis (PCA) was used to analyze these data, and score plots were used to determine whether there was discrimination between two groups [19]. Additionally, orthogonal partial least squares discriminant analysis (OPLS‐DA) was used to identify the most significant entities that caused the discrimination, with the following selection criteria: fold change > 2, *p* < 0.05, and variable influence on projection (VIP) > 1. Waters Progenesis QI 2.0 and Ezinfo 3.0 (Waters Corporation, Milford, Massachusetts, USA) were used to analyze the lipidomic data using multilevel paired multivariate statistics. The biological replicates were grouped according to whether body lotion was used. Ezinfo 3.0 was used to import the chemical information that was acquired during peak selection.

### 2.6 Cutaneous Microbiota Sampling and Analysis

To collect skin bacteria the next morning, all the participants were instructed to wash their legs the night before The participants were asked to separate body cleaning and collection by at least 8 h. The swab approach was used to collect samples from the subjects' leg skin using a sterile wetting solution (0.9% NaCl and 0.1% Tween‐20). A sterile swab was used to massage the 4 × 4 cm^2^ patches of the skin surface at least 40 times. For every sample, new sterile gloves were used to reduce cross‐contamination. For later DNA extraction, the samples were promptly stored at −80°C. DNA was extracted from skin microbiome samples utilizing a DNeasy Blood & Tissue Kit following the manufacturer's protocol (available at www.qiagen.com/handbooks). The primers and sequencing techniques employed were based on methods established in the literature [20]. The distance algorithm of principal coordinate analysis (PCoA) was the Bray–Curtis distance. The nonparametric Wilcoxon rank‐sum test was used to compare the microbial diversity between the two groups. The linear discriminant analysis (LDA) effect size (LEfSe) algorithm was used to identify significantly different species between groups (LDA scores > 3.5 k). The data were analyzed using the Majorbio I‐Sanger Cloud Platform (www.i‐sanger.com), a free online platform.

### 2.7 Statistics

The data were analyzed using SPSS software, version 26.0, and the effectiveness of the test product against the control was assessed using repeated‐measures ANOVA, paired samples *t*‐tests, and nonparametric tests, with a *p* value of less than 0.05 considered to indicate statistical significance.

## 3 Results

### 3.1 Characteristics of the Subjects at Baseline

As shown in Table 1, the study included 68 adults (26 males and 43 females), with 36 from Beijing (mean age 36.69 ± 6.05) and 32 from Shanghai (mean age 37.91 ± 4.26). The severity scores were 1.52 ± 0.50 for the treatment group and 1.54 ± 0.50 for the control group, indicating minimal differences in baseline characteristics and clinical severity between the two regions.

**TABLE 1:** Baseline characteristics of the enrolled subjects.

Group	Age mean ± SD	Sex		Dryness scores mean ± SD	
		Male	Female	Treatment	Control
Beijing	36.69 ± 6.05	18	18	1.51 ± 0.51	1.51 ± 0.51
Shanghai	37.91 ± 4.26	8	25	1.53 ± 0.51	1.56 ± 0.5
Total	37.28 ± 5.23	26	43	1.52 ± 0.50	1.54 ± 0.51

### 3.2 Body Lotion Improved the Radiance and Water Content of the Skin on the Legs

**FIGURE 1:**
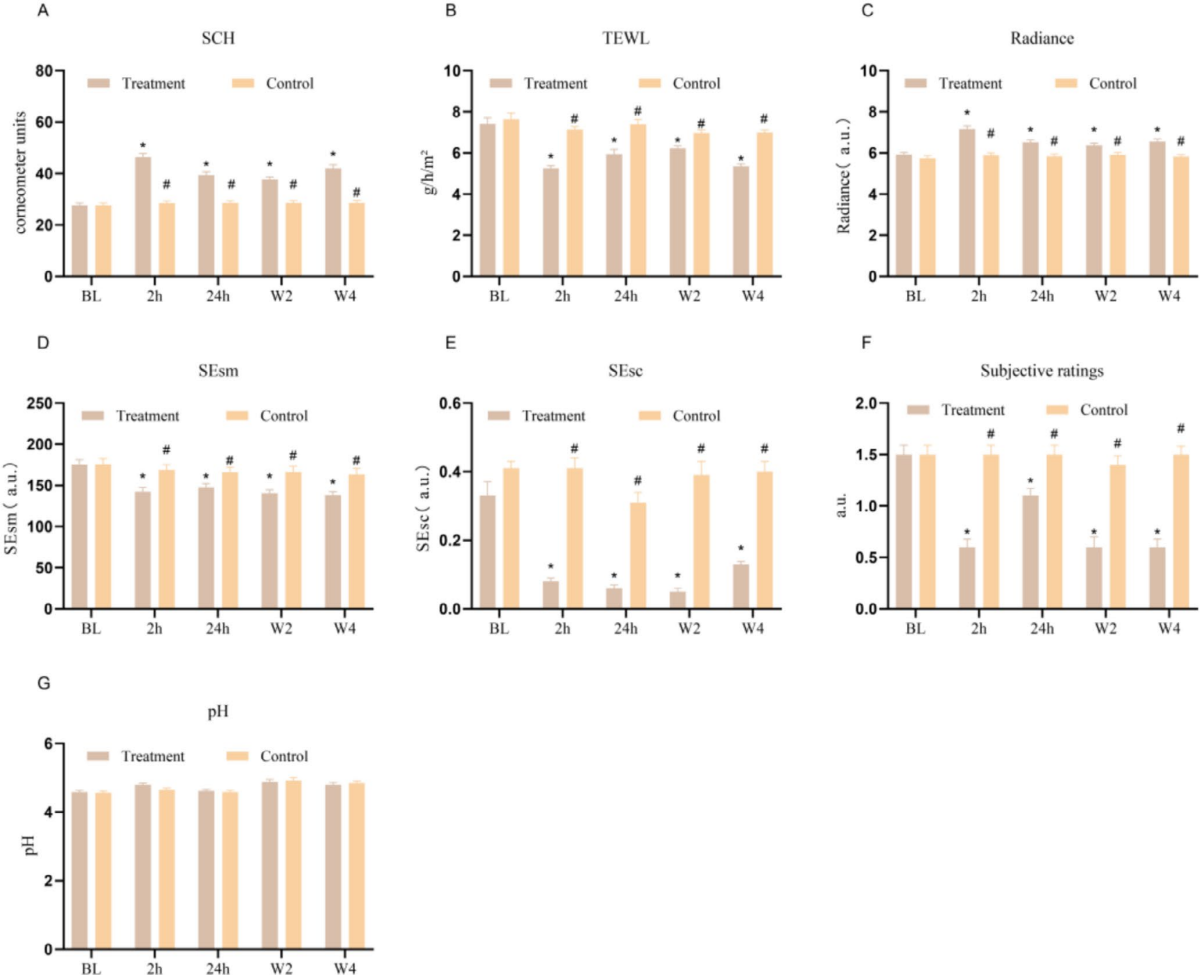
Changes in skin parameters before and after applying body lotion. Data are expressed as mean ± SD (*n* = 68), #*p* < 0.05 compared between the treatment and control groups at the same time point. *p < 0.05 compared with BL in the indicated group.

As shown in Figure 1, no skin parameters measured in the control group significantly changed relative to the baseline during the study period, and the control side had similar values to the treatment side at BL, indicating that the subjects' skin condition was stable. In contrast, compared with the baseline group, the treatment group exhibited significant increases in SCH at 2 h and 24 h postapplication. Sustained improvements were observed at W2 and W4. TEWL decreased significantly in the treatment group at 2 h, with a slight rebound at 24 h but remaining below baseline. Skin glossiness improved markedly in the treatment group at 2 h, with sustained effects through W4. Skin smoothness (SEsm) and skin scaliness (SEsc) significantly decreased from 2 h to 4 weeks after application. The subjective skin dryness score decreased quickly after intervention for only 2 h, and the change trend remained to W4. These parameters were greater in the treatment group than in the control group at all time points after the body lotion was used. However, the skin surface pH slightly increased in both groups throughout the 4 weeks.

**FIGURE 2:**
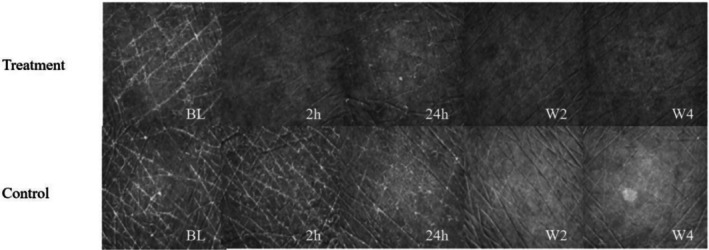
VC20plus DEMO image analyzing the effectiveness of body lotion for skin improvement.

Figure 2 displays VC20plus DEMO images documenting the changes in the skin condition of participants from both the treatment and control groups at BL, 2 h, 24 h, W2, and W4 after the product was applied. The sequence of the images indicates a reduction in skin scaling and an increase in skin smoothness after 2 weeks of product usage. The images from the fourth week continued to show improvements in skin texture on the intervention side, whereas the control side displayed persistent fine scales and lines. These findings indicate that the body lotion has good short‐ and long‐term moisturizing and barrier repair effects on dry legs.

### 3.3 Discriminant Skin Lipids Altered After Body Lotion Application

**FIGURE 3:**
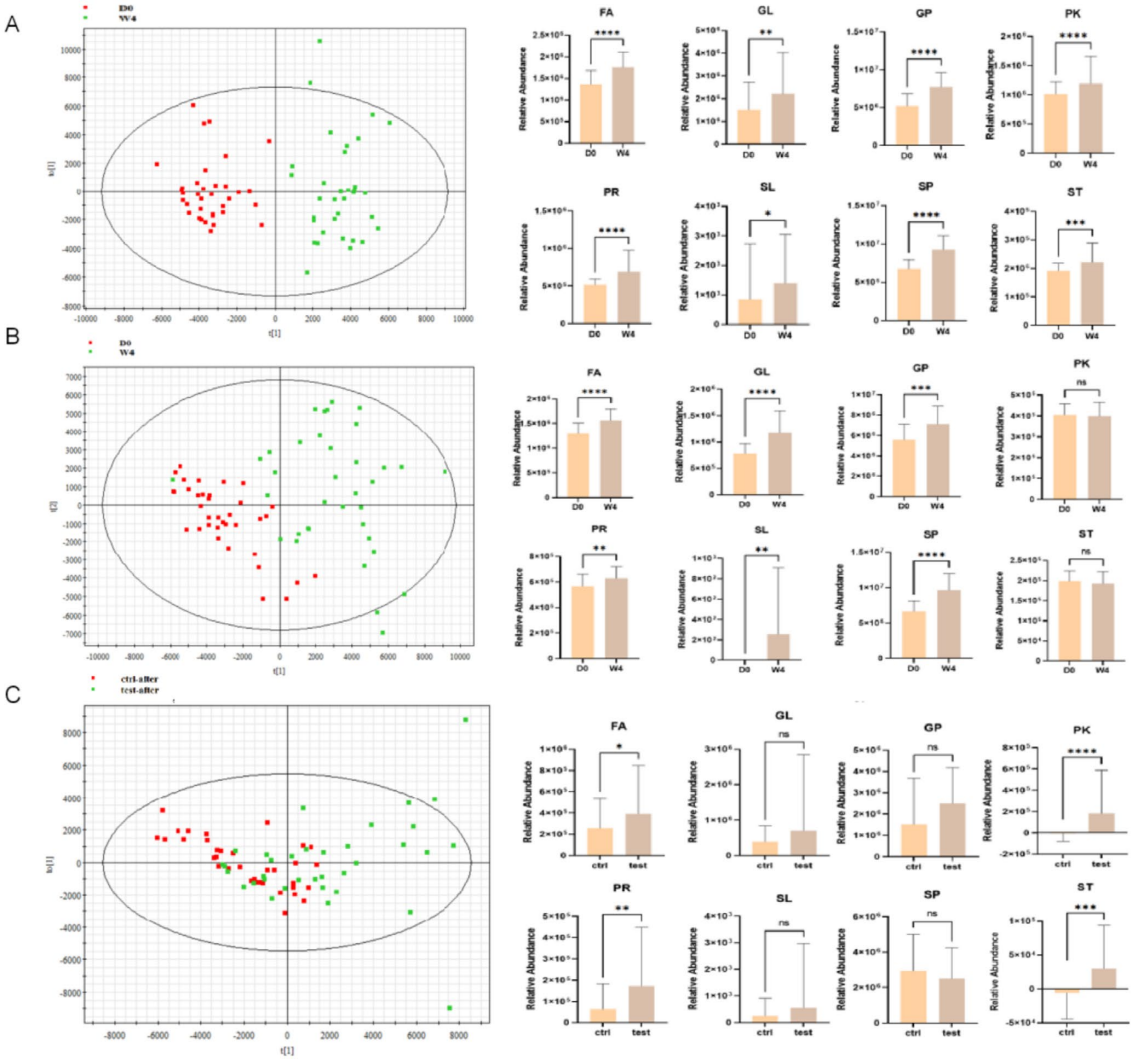
Skin lipids were altered by the body lotion use compared to baseline and control. (A) PCA score plot for the treatment group compared with the baseline and relative abundance of eight lipids within the treatment group. (B) PCA score plot for the control group compared with the baseline and relative abundance of eight lipids within the control group. (C) PCA score plot comparing the treatment and control groups following product application and relative abundance of eight lipids in the comparative analysis between the treatment and control groups post‐product application.

A total of 1267 lipids were screened and divided into eight main classes. Significant increases in the relative abundances of these eight major lipid classes—fatty acyls (FAs), glycerophospholipids (GPs), sphingolipids (SPs), sterol lipids (STs), prenol lipids (PRs), saccharolipids (SLs), and polyketides (PKs)—were observed in the treatment group compared between BL and W4, as shown in Figure 3A. However, six classes of lipids, namely, FAs, GLs, GPs, PRs, SLs, and SPs, increased in the control group after 4 weeks due to environmental interference (Figure 3B). To exclude the influence of environmental factors, we compared the lipids in the treatment group with those in the control group at W4. The relative abundances of FAs, PKs, PRs, and STs in the treatment group were dramatically greater than those in the control group (Figure 3C). These results indicate overall increases in four lipid species after 4 weeks of product use.

**FIGURE 4:**
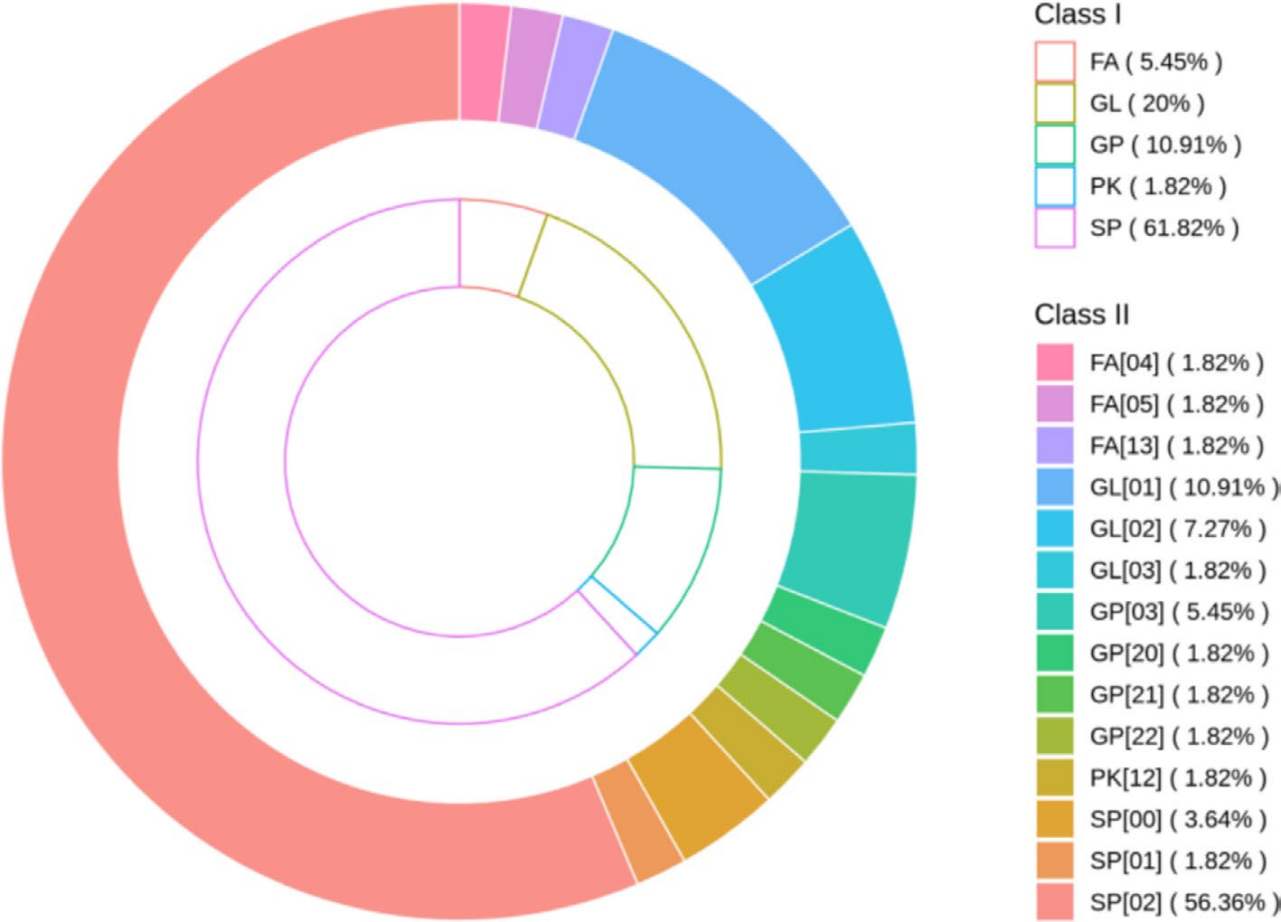
Hierarchical classification of significantly altered skin surface lipids in the treatment group. The outer ring represents the major lipid categories (Class I), and the inner ring displays the corresponding lipid subclasses (Class II). Note that SP[02] represents Ceramides, which constitute the majority of the Sphingolipids (SP) class.

The specific lipid subclasses responsible for the overall increase were identified and are presented in Table 2. Within the Sterol Lipids (ST) category, the Sterols (ST[01]) subclass exhibited significant upregulation. Furthermore, subclass‐level analysis of the sphingolipids (SPs) class revealed that ceramides (SP[02]) constituted the dominant component, representing approximately 91% of the total sphingolipids identified (Figure 4). Among these, specific ceramide subclasses—including Cer[NS], Cer[NdS], and Cer[NP]—were significantly upregulated in the treatment group. Together, these results demonstrate that the increase in total lipid content is primarily attributable to the enrichment of cholesterol and key ceramide species known to be essential for epidermal barrier function.

**TABLE 2:** List of significantly upregulated lipid subclasses in the treatment group at Week 4 compared to the control group (VIP > 1, *p* < 0.05).

Lipid category	Subclass ID	Subclass name
Sterol lipids (ST)	ST[01]	Cholesterol
	ST[02]	Steroids
	ST[04]	Bile acids and derivatives
	ST[05]	Steroid conjugates
Fatty acyls (FA)	FA[01]	Fatty Acids and Conjugates
	FA[03]	Eicosanoids
	FA[05]	Fatty alcohols
	FA[07]	Fatty esters
Prenol lipids (PR)	PR[01]	Isoprenoids
	PR[03]	Polyprenols
Polyketides (PK)	PK[12]	Flavonoids
	PK[15]	Phenolic lipids

### 3.4 Effects of Body Lotion on Skin Microbial Diversity

**FIGURE 5:**
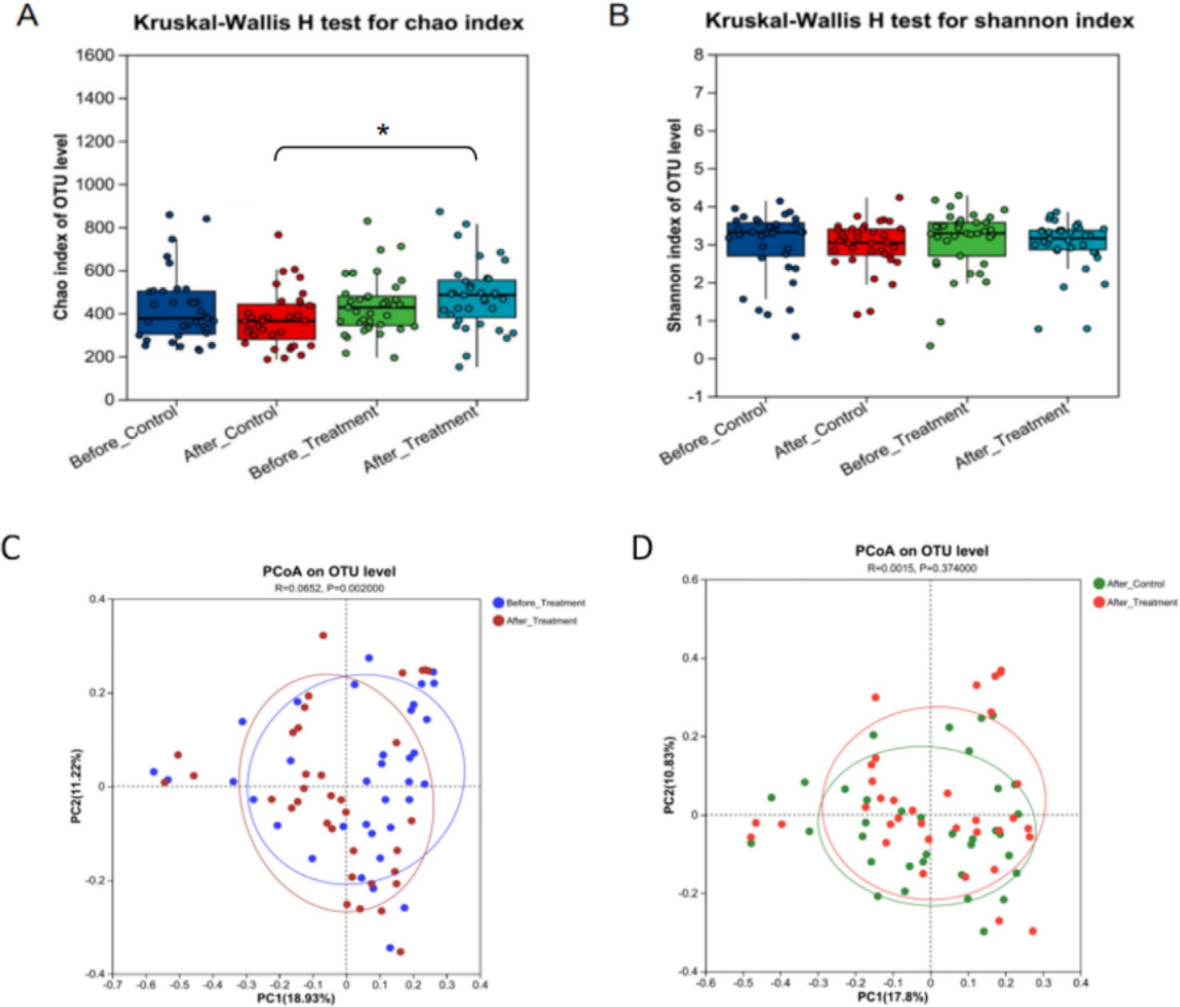
Changes in the microbiota diversity after the use of body lotion. (A, B) The distribution of Chao's index and Shannon's index in different groups.*p < 0.05 compared between the indicated groups. (C) The PCoA plots at the OTU level of the treatment group before and after the application of the product. (D) The PCoA plots at the OTU level of the control group and the treatment group.

Alpha diversity is presented as the Chao1 and Shannon indices. The Chao index increased in the body lotion group after 4 weeks of treatment compared with that in the control group (Figure 5A). However, the Shannon index remained unchanged (Figure 5B). Beta diversity was measured using Bray–Curtis dissimilarity and Jaccard similarity and was visualized by principal coordinate analysis (PCoA). The overlapping PCoA plots suggested minimal differences in beta diversity between the treatment and control groups (Figure 5C,D).

### 3.5 Body Lotion Modulated Skin Microbiota Composition at the Phylum and Genus Levels

At the phylum level, following the intervention, the relative abundance of *Firmicutes* increased from 47.16% to 49.32%, that of *Actinobacteria* decreased from 47.16% to 49.32%, and that of *Proteobacteria* decreased from 23.19% to 16.63%. Similarly, in the control group, the proportion of *Firmicutes* increased from 44.88% to 53.62%, that of *Actinobacteria* increased from 25.77% to 29.57%, and that of *Proteobacteria* decreased from 25.77% to 14.82%.

At the genus level, following the intervention, the relative abundance of *Staphylococcus* in the treatment group increased from 30.92% to 33.59%, and that of *Cutibacterium* increased from 3.32% to 8.91%. Conversely, the relative abundance of *Micrococcus* decreased from 6.32% to 4.6%. In contrast, the relative abundances of genera in the control group exhibited minimal changes.

## 4 Discussion

This study revealed that the body lotion, containing ceramides and a variety of natural oils as functional ingredients, exhibited significant moisturizing and skin‐repairing effects in subjects with xerosis, resulting in increased skin glossiness and SCH, as well as decreased TEWL, SEsm, and SEsc (Figure 1). Ceramides constitute a crucial element of the lipid matrix within the stratum corneum, facilitating the reorganization of the stratum corneum bilayer and augmenting the intercellular cohesion of keratinocytes. Consequently, this process accelerates barrier recovery, reduces TEWL, and increases SCH [21, 22]. Dry skin, marked by flakiness and a rough texture, is a key contributor to various skin issues, primarily caused by a compromised skin barrier that results in moisture loss [23]. The treatment group exhibited increased skin radiance, a smoother skin texture, and diminished scaling, as illustrated in Figure 1. Consistent with prior research, the antioxidant properties of specific plant oils may alleviate oxidative stress‐induced surface roughness of the skin, thereby enhancing its radiance [24]. In addition, the use of *Butyrospermum parkii* (shea) oil, which is rich in fatty acids and vitamins, has been demonstrated to improve skin radiance and moisture content while also promoting a smoother skin texture [25, 26]. In summary, the functional ingredients in the body lotion might be devoted to improving the biophysical properties of the skin.

Compared with baseline and control groups, the body lotion induced significant alterations in skin lipid content in the treatment group, indicating a systemic shift in skin homeostasis. Skin surface lipids (SSLs) are critical for maintaining the skin's natural protective barrier, as their components are tightly regulated in ratio between the stratum corneum (SC) and skin surface to preserve barrier integrity [27]. Given that barrier function is sensitive to changes in total lipid content or specific SSL elements, investigating SSL dynamics in xerosis is essential to elucidate daily fluctuations in barrier performance [28]. Data analysis revealed significant increases in all eight key lipid classes—FAs, GLs, GPs, SPs, STs, PRs, SLs, and PKs—with the levels of FAs, PLs, PRs, and STs showing particularly pronounced elevation relative to the control (Figure 3). The increase in GPs, lipids often associated with cutaneous microbiota [29], aligns with observed microbial shifts. Most critically, the rise in SPs, predominantly driven by Ceramide subclasses (Cer[NP], Cer[NS]), and in STs, primarily cholesterol, is central to interpreting the recovery mechanism. This increase likely results from a synergistic effect of exogenous replenishment and endogenous upregulation. Given the lotion's formulation containing ceramides (NP, NS, AP, EOP) and cholesterol, the detected enrichment supports a direct “filling” effect, supplementing deficient intercellular lipids. Concurrently, the restoration of these essential lipids may stimulate further endogenous synthesis, reinforcing the barrier's architecture [30, 31].

FAs are multifunctional: beyond energy metabolism and membrane biogenesis, they are critical for epidermal health and barrier formation, as complex lipids depend on FAs for permeability barrier integrity [32, 33]. Plant oils rich in FAs are widely used in skincare to replenish skin lipids and promote barrier repair. Evidence indicates that specific FAs and STs differentially influence skin integrity [34], and moisturizers enriched with FAs/STs enhance lipid layer flexibility and restore barrier function [35]. Our finding of concurrent increases in FAs, SPs, and STs is consistent with studies demonstrating that moisturizers supplemented with such oils improve lipid profiles in barrier‐impaired skin [9], implying a potentially more pronounced effect in xerosis characterized by significant lipid depletion.

The observed lipidomic changes hold distinct biological relevance. Restoration of SLs, the cornerstones of the SC barrier, directly explains improved hydration and reduced TEWL [31]. Simultaneously, the modulation of PKs—primarily microbial metabolites—provides biochemical evidence of functional microbiome remodeling [36], suggesting commensal flora participate in homeostasis via bioactive secretion. Furthermore, optimized PRs may enhance the skin antioxidant defense system [37]. Collectively, the synchronized recovery of host‐derived barrier lipids (SLs, STs) and microbiome‐associated metabolites (PKs) underscores the concept of beneficial lipid‐microbiome crosstalk in alleviating xerosis.

**FIGURE 6:**
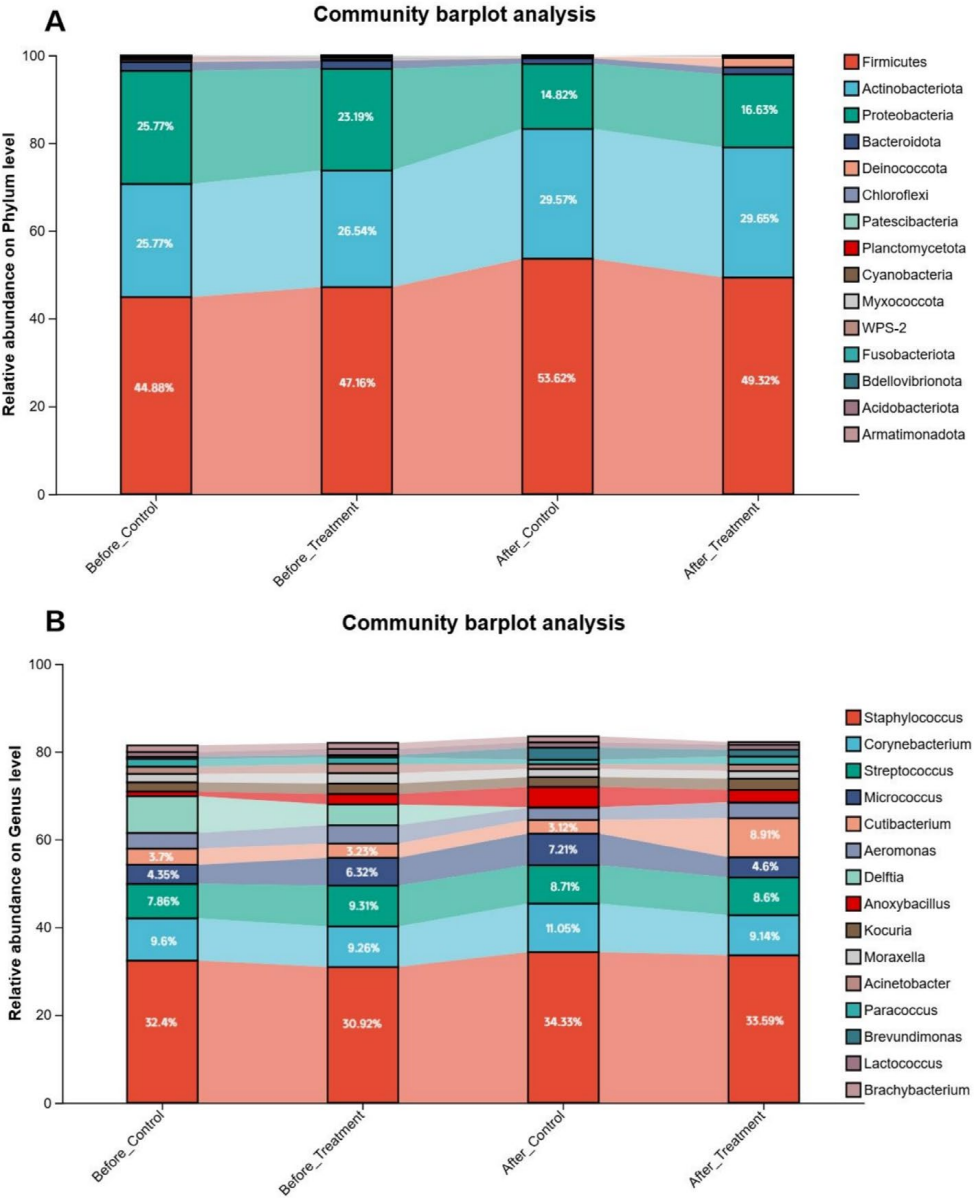
The body lotion affected the relative abundance of differential taxa at the genus and phylum levels. (A) Relative abundance of microbial communities at the phylum level. (B) Relative abundance of microbial communities at the genus level.

**FIGURE 7:**
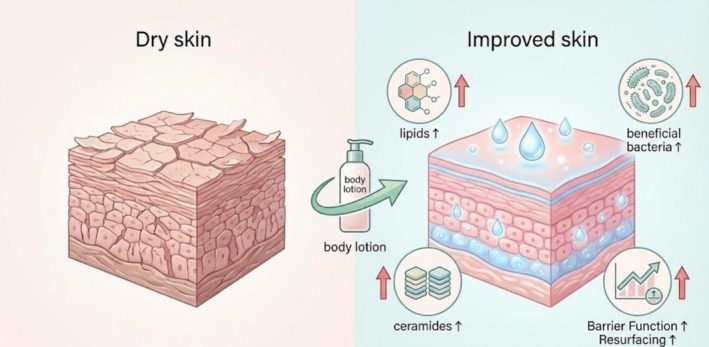
Schematic diagram of the proposed mechanism for the body lotion in alleviating xerosis.

The Chao index, which represents the alpha diversity of the skin microbiome, increased with the application of the body lotion (Figure 5). These findings are consistent with previous research involving the use of a moisturizer, which resulted in increased bacterial diversity [38]. At the phylum level, there were notable increases in the abundances of *Cutibacterium* and *Firmicutes* following treatment, whereas the abundance of *Proteobacteria* decreased (Figure 6A). At the genus level, the relative abundances of *Staphylococcus* and *Corynebacterium* increased, and the relative abundance of *Micrococcus* decreased (Figure 6B). *Staphylococcus* species are skin‐resident bacteria that are mostly found in moist skin regions [38]. Elevated *Staphylococcus* levels indicate that the body lotion moisturizes the skin and may encourage increased SCH, which further implies that there is a moisturizing effect on the skin with the use of the body lotion [39]. Furthermore, a study demonstrated that individuals with dry skin presented increased skin lipid content following the application of an emollient, as well as a notable increase in the presence of *Cutibacterium*, which may be attributed to its propensity to proliferate in lipid‐rich environments [40, 41]. This increase in microbial populations suggests an amelioration of skin conditions, as *Cutibacterium* plays a crucial role in maintaining skin pH and modulating immune responses. The ability of the lotion to increase lipid levels further supports the equilibrium of the skin's natural sebum–microbe interface [42]. These findings suggest that the product has the ability to reduce skin barrier damage, improve the health of the skin of patients with xerosis and balance the skin microecology to a stable state. The potential mechanism by which the body lotion alleviates xerosis through the modulation of lipid metabolism and microbial community is illustrated in Figure 7.

## 5 Conclusions

In summary, this study systematically evaluated the moisturizing and skin‐repairing effects of a body lotion formulated with ceramides and natural oils in xerosis subjects, which alleviated skin dryness and scaliness, restored barrier function, and enhanced radiance and smoothness. Mechanistically, these benefits arise from synergistic regulation of skin lipid homeostasis and microbiome dynamics, with multi‐omics analysis revealing correlations between these microenvironmental changes and barrier repair. These findings provide multi‐dimensional mechanistic evidence—spanning biophysical, lipidomic, and microbiological dimensions—for the efficacy of a functional moisturizer, offering a scientific rationale for integrated skincare strategies that target both barrier repair and microenvironment balance. Future research should focus on the long‐term maintenance of these benefits and the causal pathways underlying the observed lipid‐microbiome crosstalk to guide the development of targeted interventions.

## Author Contributions

Conceptualization: Jie Yang and Jun Wang. Methodology: Lu Cheng and Yi Qin. Software: Fengwei Qi and Fan Wu. Investigation: Qitian Fu and Xiaofeng Bai. Writing: Jun Wang and Jiaqi Zhang. Supervision: Yao Pan. All authors have read and agreed to the published version of the manuscript.

## Funding

This research did not receive any specific grant from funding agencies in the public, commercial, or not‐for‐profit sectors.

## Ethics Statement

The clinical research protocol involving human subjects was followed and authorized by the Shanghai Ethics Committee (Approval No. SECCR2024‐128‐01).

## Consent

Each participant provided written informed consent before participation.

## Conflicts of Interest

The authors declare no conflicts of interest.

## Data Availability

The data that support the findings of this study are available on request from the corresponding author. The data are not publicly available due to privacy or ethical restrictions.
